# Superstitions on Human Papillomavirus in Africa: A Scoping Review

**DOI:** 10.1002/puh2.70255

**Published:** 2026-05-21

**Authors:** Jimoh Amzat, Kafayat Aminu, Rita Amarachi Nwebo, Precious Nnannah, Chiamaka Norah Ezeagu, Ganiyat Amzat, Kehinde Kazeem Kanmodi

**Affiliations:** ^1^ Department of Sociology Usmanu Danfodiyo University Sokoto Nigeria; ^2^ Department of Sociology University of Johannesburg Johannesburg South Africa; ^3^ School of Health and Life Sciences Teesside University Middlesbrough UK; ^4^ Centre for Evidence Synthesis and Implementation Research Cephas Health Research Initiative Inc Ibadan Nigeria; ^5^ Department of Economics Usmanu Danfodiyo University Sokoto Nigeria; ^6^ Office of the Executive Director Cephas Health Research Initiative Inc Ibadan Nigeria; ^7^ Department of Public Health Thomas Adewumi University Oko Nigeria; ^8^ Department of Research and Innovation University of Technology and Entrepreneurship Phnom Penh Cambodia

**Keywords:** Africa, human papillomavirus (HPV), misconceptions, superstitions, vaccine hesitancy

## Abstract

**Background:**

Human papillomavirus (HPV) is a leading cause of cervical cancer, with Africa bearing a significant disease burden. Despite the availability of effective vaccines, superstitions and misinformation hinder HPV vaccine uptake, contributing to high mortality rates. This scoping review examines superstitious beliefs and misconceptions surrounding HPV and its vaccine in Africa, their cultural and social underpinnings, and their impact on public health interventions.

**Methods:**

Following the Arksey and O'Malley framework, this review adhered to the Preferred Reporting Items for Systematic Reviews and Meta‐Analyses for Scoping Reviews (PRISMA‐ScR) guidelines and systematically analyzed literature obtained from nine databases. Seventeen studies, involving 2516 participants from seven African countries, met the inclusion criteria. We analyzed the data using thematic synthesis, and patterns such as superstitions, community narratives, and vaccine hesitancy were identified.

**Results:**

Findings indicate widespread misconceptions, including beliefs that HPV is caused by curses, exposure to sunlight, or spiritual forces. Superstitions that link HPV vaccination to infertility, chronic diseases, and depopulation conspiracies are prevalent. Traditional and religious leaders played a critical role in shaping public perception, perpetuating mistrust in HPV vaccines.

**Conclusion:**

Superstitions reduce HPV vaccine acceptance and increase cervical cancer risks. Targeted health education, community engagement, and culturally sensitive interventions are crucial to dispelling myths and improving vaccine uptake in Africa.

## Graphical abstract

Africa bears a high burden of HPV‐induced diseases; this scoping review explored how superstitions and cultural beliefs fuel vaccine hesitancy. There are widespread myths and opposition from cultural/religious leaders that drive HPV vaccine hesitancy; targeted and culturally sensitive campaigns/educations are essential to improve vaccine uptake and reduce cervical cancer burden.

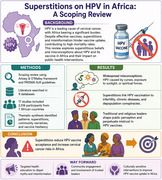

## 1 Background

Human papillomavirus (HPV) is one of the most prevalent sexually transmitted infections (STIs) worldwide and is a significant public health concern due to its association with cervical cancer and other anogenital malignancies [1, 2]. HPV is responsible for approximately 99% of cervical cancer cases globally, with Africa bearing a disproportionately high burden of the disease [3, 4]. Sub‐Saharan Africa, in particular, records some of the highest cervical cancer mortality rates due to limited access to preventive healthcare, screening, and treatment services [4]. Although HPV vaccines, such as Gardasil and Cervarix, have proven to be highly effective in preventing HPV infections and related cancers, their uptake in many African communities remains low [3]. This is partly due to misinformation and superstitions that fuel fear, stigma, and resistance to vaccination and treatment. Despite advancements in HPV prevention and treatment, including vaccination and screening programs, many African communities continue to hold deep‐rooted superstitious beliefs about the virus. These superstitions influence health‐seeking behavior, vaccine acceptance, and general public perception of HPV, contributing to the persistent burden of HPV‐related diseases in Africa [5, 6].

In many African societies, cultural and social beliefs also play a crucial role in the low uptake of the vaccine [7]. This belief system hinders timely diagnosis and treatment, worsening HPV‐related health outcomes. Misinformation, sometimes spread through religious and cultural institutions, discourages parents from allowing their daughters to receive the vaccine, leaving them vulnerable to HPV infection and subsequent cervical cancer. Generally, health beliefs play a significant role in shaping health behaviors, health‐seeking patterns, and overall public health outcomes in Africa [8]. Traditional, religious, and cultural beliefs often influence perceptions of disease, treatment, and prevention, sometimes leading to poor health outcomes [9]. Many communities still rely on traditional medicine, faith healers, and spiritual interventions, which can delay or replace evidence‐based medical treatments, particularly for conditions such as HIV/AIDS, malaria, and cancer [10]. Misinformation and superstitions about vaccines, reproductive health, and infectious diseases have also led to widespread vaccine hesitancy. For instance, rumors linking polio and HPV vaccines to infertility have hindered immunization efforts in several African countries, posing a significant public health challenge [11].

Although studies have explored HPV knowledge and vaccine hesitancy in Africa, limited research has systematically analyzed the role of superstitions and cultural beliefs in shaping public perceptions of the virus. Understanding these superstitions is crucial in designing culturally sensitive interventions that address misinformation and increase vaccine uptake. This scoping review seeks to examine the various superstitions surrounding HPV in Africa, their cultural underpinnings, and their impact on public health interventions and recommend strategies to counteract misinformation. By examining the intersection of superstitions and HPV in Africa, this study will contribute to the broader discourse on health literacy, cultural beliefs, and infectious disease prevention.

## 2 Methods

### 2.1 Study Design

This study followed a scoping review methodology based on the Arksey and O'Malley framework, a widely recognized approach for conducting such reviews [12]. The primary aim of this review was to map existing literature on superstitions related to HPV in Africa. The best practice checklists, PRISMA‐ScR (Preferred Reporting Items for Systematic Reviews and Meta‐Analyses extension for Scoping Reviews) and AMSTAR 2 (A Measurement Tool to Assess Systematic Reviews, Version 2), were adhered to in this review [13]. These checklists were employed to ensure adherence to reporting standards [14].

### 2.2 Identification of the Research Question

The research question guiding this scoping review was as follows: “What are the superstitions related to HPV in Africa, and how do they influence health‐related behaviors and outcomes?” To structure this research question, we utilized the PCC format. The population of interest (P) was individuals and population groups in Africa of any sociodemographic background. The concept (C) was superstitious beliefs. The context (C) was HPV.

### 2.3 Identification of the Relevant Studies

**FIGURE 1:**
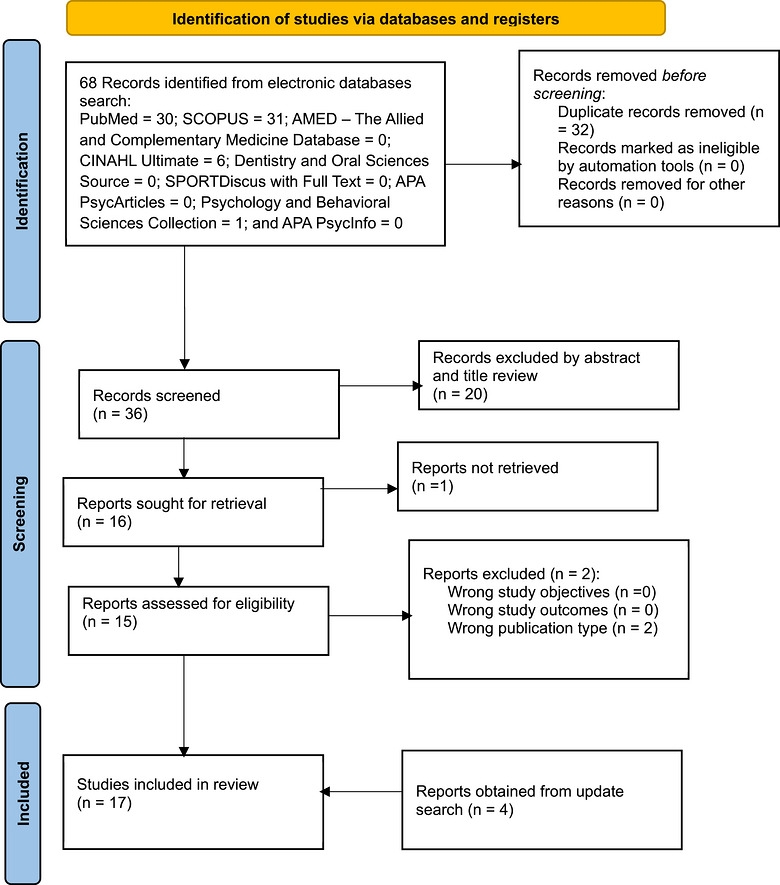
PRISMA flow diagram highlighting the included studies in compliant with PRISMA 2020 guidelines.

On December 9, 2024, a search across nine electronic research databases to gather all studies, published from inception till date, that are relevant to the superstitions of HPV in Africa was systematically conducted. The databases searched included SCOPUS, PubMed, CINAHL Ultimate, APA PsyArticles, SPORTDiscus with Full Text, The Allied and Complementary Medicine Database (AMED), Dentistry and Oral Sciences Source, Psychology and Behavioral Sciences Collection, and APA PsycInfo. The systematic search utilized terms such as “superstit*,” “fallacy,” “delusion,” “misconception,” “fantasy,” “falsehood,” “falsity,” “false notion,” “irrational belief,” “human papilloma,” “HPV,” and specific country names in Africa like “Algeria,” “Angola,” “Benin,” “Botswana,” “Burkina Faso,” “Burundi,” “Cameroon,” “Central African Republic,” “Chad,” “Ivory Coast,” “Cote d'Ivoire,” “Djibouti,” “Democratic Republic of Congo,” “Egypt,” “Equatorial Guinea,” “Eritrea,” “Eswatini,” “Ethiopia,” “Gabon,” “Gambia,” “Ghana,” “Guinea,” “Guinea‐Bissau,” “Kenya,” “Lesotho,” “Liberia,” “Libya,” “Madagascar,” “Malawi,” “Mauritania,” “Mauritius,” “Mali,” “Mauritania,” “Morocco,” ”Mozambique,“ “Namibia,” “Niger,” “Nigeria,” “Rwanda,” “Sao Tome and Principe,” “Senegal,” “Seychelles,” “Sierra Leone,” “Somalia,” “South Africa,” “Sudan,” “Tanzania,” “Togo,” “Tunisia,” “Uganda,” “Zambia,” “Zimbabwe,” “Reunion,” “Saint Helena,” “Western Sahara,” and “Mayotte.” Boolean operators “OR” and “AND” were used to combine the search terms and refine the search results. The search strings used for each database search are provided in Tables S1‐S3. Moreover, an update search was done on December 16, 2025, using these search strings to manually identify any new literature that has been published after the initial literature search date (Figure 1).

### 2.4 Selection of Relevant Studies

To manage the selection process, the Rayyan web application was used to identify and remove duplicate records from the collected literature. After de‐duplication, the remaining articles were thoroughly screened to assess their eligibility for inclusion in the review. To be included in this review, the screened literature needed to meet several criteria. The literature reports empirical findings specifically addressing superstitions related to any health‐related issue concerning HPV and adopts any research design (quantitative design, qualitative design, or mixed‐methods design). Only peer‐reviewed journal articles were considered to maintain a high standard of academic rigor and reliability. The articles needed to be published in English (i.e., because none of the reviewers could speak an international academic language other than English). Additionally, the full text of the articles had to be accessible, allowing for a detailed review and data extraction. Articles that did not meet these eligibility criteria were excluded from the study.

The screening process involved a thorough examination of titles, abstracts, and keywords, by at least two reviewers (K.A., R.A.N., and P.N.) to preliminarily identify articles that appeared to meet the inclusion criteria. Following this initial screening, the full texts of potentially eligible articles were retrieved and reviewed in detail to confirm their suitability for inclusion. This two‐stage screening process ensured that only the most relevant articles were included in the final review. R.A.N. and P.N. conducted each stage of the screening independently. Any disagreements regarding the inclusion or exclusion of studies were resolved by consulting K.A. who was the third reviewer. Senior experts on the research team conducted a review of the included articles (J.A. and K.K.K.), and any inconsistencies were addressed collectively by the entire team. However, there were minimal conflicts during this process, all of which were resolved.

### 2.5 Quality Appraisal

The methodological quality of the studies incorporated in this review was evaluated using the Mixed Methods Appraisal Tool (MMAT), version 2018 [15]. This tool is structured with two core questions applicable to all research designs, followed by an additional five questions tailored to the specific methodological approach of each study—namely, qualitative, quantitative (including randomized controlled trials, non‐randomized, and descriptive designs), and mixed methods.

To maintain a rigorous and transparent appraisal process, two reviewers (K.K.K. and K.A.) independently assessed each study. Evaluations were conducted using a three‐point scale, where “yes” was assigned a score of 1, “can't tell” a score of 0.5, and “no” a score of 0. These scores were then summed to yield an overall quality rating for each study, ranging from 0 to 7. Studies were subsequently categorized as “below average” (score < 3.5), “average” (score = 3.5), or “above average” (score > 3.5) based on this total.

Any discrepancies between the two primary reviewers were addressed through discussion. In instances where consensus could not be reached, a third reviewer (J.A.) was consulted to arbitrate and confirm the final quality assessment. The complete results of this appraisal are provided in Tables S5–S9.

### 2.6 Data Charting

Using a structured approach, relevant information was methodically extracted from the included articles. A bespoke charting form was developed to ensure consistency and comprehensiveness. Key elements charted from each study included study details (title, authors, year of publication, journal), objectives, methodology (study design, sample size, population characteristics, data collection methods), key findings related to superstitions on HPV (cultural beliefs, health outcomes, access to services), geographical focus, and conclusion (see Table 1). The data charting form was pilot‐tested on a subset of studies to ensure its adequacy and comprehensiveness, with adjustments made based on feedback from the research team. Once finalized, the form was used for systematic data extraction from all included studies.

**TABLE 1:** Studies on superstitions about human papillomavirus (HPV) in Africa.

No.	Author(s) and year	Research objective(s)	Study design	Country	Setting (rural/urban)	Sample size	Participants	Type of intervention to address superstitions on HPV vaccine	Data collection method	Results related to the review objective(s) and question(s)	Conclusion/Recommendation
1.	Bitariho et al. (2023) [19]	To assess knowledge, perceptions, and practices of girls (10–14 years) in Uganda and to produce evidence to guide HPV vaccine uptake programs	Mixed‐methods design	Uganda	Urban and rural	524 (quantitative component = 524; qualitative component = total not specified (6 focus group discussions, and 24 key informant interviews))	Adolescent girls (10–14 years); teachers and parents	None	Convergent parallel mixed‐methods design	Negative beliefs, superstitions, and safety concerns among parents that may impact their willingness to have their daughters vaccinated were noted	It was concluded that all stakeholders should be sensitized about HPV vaccine
2.	Bwanali, et al. (2024) [21]	To identify barriers to caregiver acceptance of HPV vaccine for their female children and establish the consequential willingness to vaccinate their children	Qualitative study design	Malawi	Semi‐urban	50 (male‐16; female‐34)	Caregivers (married and single) with at least 1 female child aged 9–14 years	None	Focus group discussions	Widespread superstitions about HPV vaccine like the government's scheme to make girls infertile to control the population hinder its uptake	Notwithstanding the awareness campaign prior HPV immunization drive, misconceptions mar HPV vaccine uptake
3.	Kaaria et al. (2024) [22]	To determine religious leaders’ willingness to promote HPV vaccine uptake in Mavoko Sub‐County, Machakos County, Kenya	Quantitative analytical study	Kenya	Peri‐urban	198 (male: 102 and female: 96)	Religious leaders	None	Survey	The majority of respondents did not believe that HPV infection affects those involved in immoral behavior and that they deserve their condition	Although there were concerns and misconceptions, most participants expressed a willingness to support vaccination
4.	Turiho et al. (2014) [26]	To assess girls’ knowledge of cervical cancer and HPV vaccine, and their acceptance of future vaccination of friends and hypothetical daughters	Mixed‐methods study	Uganda	Rural and urban	777 girls (Ibanda‐444 and Mbarara‐333) (quantitative = 777, qualitative = not specified) (five FGDs each with 8–12 vaccinated girls)	Primary school girls (vaccination cohorts)	Pre‐vaccination sensitization	Survey and focus group discussions	Childbirth complications, infertility, and mysterious conception of twins are some of the superstitions recorded among Ugandan school girls	Attitudes toward HPV vaccine was determined by multiple factors other than knowledge
5.	Wubu et al. (2023) [29]	To explore the perception of secondary school girls toward HPV vaccine in Ethiopia	Qualitative (phenomenological) study design	Ethiopia	Sub cities	58 female students	Middle adolescent secondary school girls (14–17 years)	None	Focused group discussions	Misconceptions, myths, lack of credible information sources, and cultural and religious perspectives were hindrances to HPV vaccine uptake	HPV vaccine uptake can be enhanced through collaborations with stakeholders to address the challenges identified
6.	Agyei‐Baffour et al. (2020) [17]	To examine Ghanaian healthcare providers’ attitudes toward HPV vaccination and their vaccination recommendation practices	Qualitative study	Ghana	Urban	29 (15 male and 14 female)	Healthcare providers (29 and 42 years)	None	Focus group discussions	HPV vaccine is associated with immorality; hence, its uptake is affected by religious objection and social stigma	The need for comprehensive HPV education focusing on vaccine efficacy and addressing stigma was emphasized
7.	Venturas and Umeh (2017) [28]	To explore health professionals’ perspectives on the HPV vaccination program in Zambia	Qualitative study	Zambia	Urban	15 (4 males, 11 females)	Healthcare professionals	None	Semi‐structured interviews and observations	Participants indicated that there are widespread myths and beliefs about HPV vaccine that created fear in the community	Health professionals need to acknowledge their misconceptions about HPV vaccination and address community resistance
8.	Balogun and Omotade (2018) [18]	To explore contextual interpretations of stakeholders regarding cervical cancer and HPV vaccines for adolescents in five selected communities in Ibadan	Qualitative study	Nigeria	Urban	190 (male‐110; female‐80)	Adolescents, parents of adolescents, religious and traditional leaders, teachers, and traditional healers	None	Focus group discussions	Belief that civilization, change in lifestyle, and promiscuity are major causes of cervical cancer rather than HPV was widespread	Myths and misconceptions about HPV and cervical cancer should be corrected for HPV vaccine for adolescents to have a positive outcome
9.	Brandt et al. (2019) [20]	To explore perceptions and acceptability of HPV self‐sampling‐based cervical cancer screening and to identify preferences and socio‐cultural barriers regarding self‐sampling	Qualitative study	Ethiopian	Rural	41 (20 and 38 years)	Sexually active women (20–65 years)	None	Focus group discussion	Myth that Mitat/Girefat (sudden exposure of the body to sunlight) is the cause of cervical cancer was common	Existing social networks are important in disseminating information on health issues, including HPV self‐sampling
10.	Lubeya et al. (2023) [23]	To understand adolescent girls’ knowledge and perceptions regarding the HPV vaccine and discuss its acceptability and uptake implications	Qualitative study	Zambia	Urban	30	Adolescent girls aged 15–18 years	None	Semi‐structured interviews	Widespread myths, misinformation, and misconceptions hinder HPV vaccine uptake	To enhance HPV vaccine acceptance and uptake among adolescent girls in Zambia, it is essential to actively engage key stakeholders and dispel myths and misconceptions surrounding the vaccine
11.	Ochomo et al. (2024) [24]	To understand the concerns and misconceptions surrounding HPV vaccine	Qualitative study	Kenya	Rural area	48 (24 adolescents and 24 parents)	Adolescent girls (aged 10–14) and their parents Both vaccinated and unvaccinated girls	None	Focused group discussions	Different myths and misconceptions about HPV vaccine were reported, including effects on fertility, unexplained illness, and conspiracy to depopulate Africa	Strategic communication and collaborative efforts are important for improving HPV vaccine coverage
12.	Rujumba et al. (2021) [25]	To explore barriers that prevent eligible girls from initiating or completing the recommended 2‐dose HPV vaccine series	Qualitative study	Uganda	Rural	40	Primary school girls, their caregivers, healthcare workers, Village Health Team members (VHTs) and teachers or school administrators	None	In‐depth interviews and key informant interviews	Rumors, misconceptions, strong traditional and religious beliefs about HPV and vaccination were common and discouraged people from getting vaccinated in the community	Accurate information to debunk rumors and misinformation in the community, school, and health facility will promote a positive attitude toward HPV vaccine
13.	Turiho et al. (2017) [27]	To explore community member's perceptions about HPV vaccination and implications for acceptability of HPV vaccination	Qualitative study design	Uganda	Sub‐counties	105 (43 schoolgirls; 52 parents; 3 health workers; 2 community leaders; and 5 teachers)	Schoolgirls (13–16 years); parents/guardians; teachers; health workers; and community leaders	None	Focus group discussions and key informant interviews	Some pestering concerns and misconceptions about HPV vaccination affecting its acceptability were identified	Initial rumors, fears, and concerns that discouraged HPV vaccination had waned, hence positive attitude to vaccination
14.	Mhango et al. (2025) [31]	To assess the acceptability, feasibility, and appropriateness of integrating HPV self‐sampling for cervical cancer screening (CCS) into family planning (FP) services	Mixed‐methods design	Malawi	Urban, peri‐urban, and rural health facilities and communities	273 healthcare providers (quantitative = 273 qualitative = not specified)	Healthcare providers: nurses, clinicians, laboratory staff, Health Surveillance Assistants (HSAs), facility managers	HPV vaginal self‐sampling; integration of cervical cancer screening into family planning services; and community education	Quantitative surveys; in‐depth interviews (IDIs); focus group discussions (FGDs)	Self‐sampling reduced fears and dispelled myths and misconceptions related to cervical cancer screening and family planning methods	Integrating HPV self‐sampling into family planning services is acceptable, feasible, and appropriate in LMIC settings
15.	Makanani et al. (2025) [32]	To explore barriers to HPV vaccine uptake and identify strategies to improve vaccination among female adolescents living with HIV and their guardians	Qualitative (phenomenological) study design	Malawi	Rural and urban	30	Female adolescents living with HIV; caregivers; healthcare workers	Education and sensitization, caregiver counseling, provider recommendations, community awareness, and integration of HPV vaccination into teen clubs	Focus group discussions and in‐depth interviews	Superstitions and misconceptions were major barriers. Negative parental attitudes, inadequate HPV information, and vaccine stock‐outs reduced uptake Trusted provider recommendations, caregiver education, and teen‐club‐based delivery were identified as effective strategies to counter misinformation	Regular health education for caregivers and adolescents, healthcare provider recommendations, reliable vaccine availability, and integration of HPV vaccination into teen clubs are recommended to dispel superstitions and improve uptake among adolescents living with HIV
16.	Abrefah et al. (2025) [33]	To examine the perspectives of Ghanaian adolescent students on HPV vaccination and its role in preventing cervical cancer	Qualitative study design	Ghana	Semi‐urban and urban	59	Adolescent students	None	Focus group discussions	There was widespread misconception, such as HPV causes infertility or “destroying the womb.” These served as barriers to HPV vaccination	Urgent need for culturally relevant, school‐based HPV education programs in Ghana to address misconceptions and improve vaccine uptake
17.	Manga et al. (2025) [30]	To identify and rank predominant HPV vaccine misconceptions in Nigeria, summarize the stability of expert judgments and translate the prioritized list into communication and training recommendations	Quantitative descriptive study	Nigeria	Rural and urban areas	49	Healthcare professionals	None	Desk review of media; validation meetings; and Delphi surveys	Several misconceptions were identified and ranked, including HPV vaccine causes infertility/population control; promotes sexual promiscuity; western conspiracy/unknown long‐term side effects	Most popular misconceptions should be addressed through staged communication: collaborations with religious/cultural leaders and sustained assessments recommended

Microsoft Word document was utilized for the data extraction process, taking advantage of its familiar and user‐friendly interface to efficiently organize and document the information. Any discrepancies or uncertainties encountered were carefully resolved through detailed discussions and consensus among the reviewers and a senior member of the team, J.A., a professor of sociology.

### 2.7 Collation, Summary, and Reporting of Results

In this review, the narrative synthesis approach was used to identify and report patterns within the extracted data [16]. This approach allowed for the organization of the extracted information into coherent themes, providing a detailed understanding of superstitious beliefs about HPV and their impact on African communities. Initially, the data were reviewed multiple times to identify recurring topics and significant points. Relevant data were coded and labeled with tags that described their content. These codes were then grouped into broader categories reflecting significant themes or patterns. The themes were reviewed and refined to ensure they accurately represented the data and were distinct from one another. The identified themes were cross‐checked by multiple research team members to ensure validity and reliability. The final themes were described in detail, supported by direct quotes and specific examples from the data. No conflict was recorded throughout this process.

## 3 Results

Figure 1, which is the PRISMA flow diagram, outlines the systematic process of identifying, screening, and including studies in the scoping review on superstitions about HPV in Africa. A total of 68 records were retrieved from the initial search (dated December 9, 2024) of electronic databases (PubMed, SCOPUS, CINAHL Ultimate, and others), with 32 duplicates removed, leaving 36 records for title and abstract screening. After screening, 20 records were excluded due to irrelevance, and the remaining 16 records were assessed for full‐text eligibility. One full text could not be retrieved, and two additional records were excluded at this stage because they were wrong publication types, resulting in 13 peer‐reviewed empirical journal articles being included from the initial search. On December 16, 2025, an update search was conducted, from which four new eligible articles were obtained. Finally, a total of 17 articles were included in this scoping review. The diagram follows PRISMA 2020 guidelines, ensuring transparency and methodological rigor in selecting relevant literature for analysis.

**FIGURE 2:**
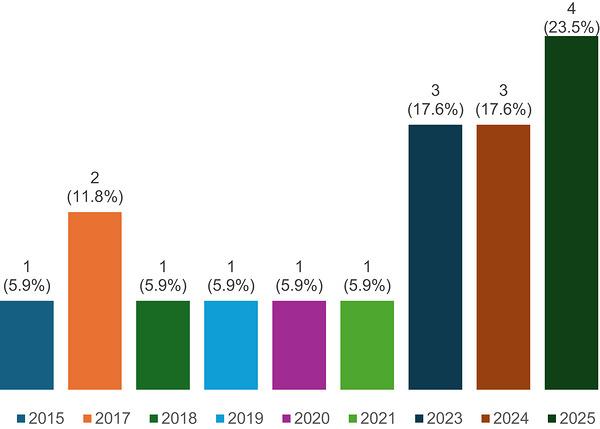
Distribution of included studies by year of publication.

In total, only 17 articles [17, 18, 19, 20, 21, 22, 23, 24, 25, 26, 27, 28, 29, 30, 31, 32, 33] met the criteria for inclusion, indicating low research focus on the topic across the region. As Figure 2 depicts, a total of seven articles reported superstitions on HPV in Africa between 2015 and 2021. There was a sharp increase in the number of articles on the topic between 2021 and 2025.

### 3.1 Sample, Study Design, and Data Collection Approach

Seventeen eligible articles were included in the current scoping review, involving 2516 participants, with females constituting 82.7% of the sample (Tables 1, 2, 3). A significant proportion of the reviewed studies (12 out of 17) utilized qualitative research design, adopting phenomenological, narrative, and interpretative methods in their research [17, 18, 20, 21, 23, 24, 25, 27, 28, 29, 32, 33]. Three utilized mixed‐methods design [19, 26, 31], whereas two utilized quantitative study design, including analytical and descriptive design [22, 30].

**TABLE 2:** Breakdown of included studies by sample size and gender of participants.

Author(s) and year	Sample size	Gender	
		Male	Female
Bitariho et al. (2023) [19]	524	0	524
Bwanali et al. (2024) [17]	50	16	34
Kaaria et al. (2024) [18]	198	102	96
Turiho et al. (2014) [19]	777	0	777
Wubu et al. (2023) [20]	58	0	58
Agyei‐Baffour et al. (2020) [21]	29	15	14
Venturas and Umeh (2017) [22]	15	4	11
Balogun and Omotade (2018) [23]	190	110	80
Brandt et al. (2019) [24]	41	0	41
Lubeya et al. (2023) [25]	30	0	30
Ochomo et al. (2024) [26]	48	0	48
Rujumba et al. (2021) [27]	40	0	40
Turiho et al. (2017) [28]	105	0	105
Mhango et al. (2025) [31]	273	145	128
Makanani et al. (2025) [32]	30	3	27
Abrefah et al. (2025) [33]	59	9	50
Manga et al. (2025) [30]	49	30	19
**Total**	**2516**	**434**	**2082**

**TABLE 3:** Breakdown of studies by sample size and study type.

Author(s) and year	Study type		
	Qualitative	Quantitative	Mixed‐methods
Manga et al. (2025) [30]		49	
Kaaria et al. (2024) [22]		198	
Turiho et al. (2014) [26]			777
Mhango et al. (2025) [31]			273
Bitariho et al. (2023) [19]			524
Makanani et al. (2025) [32]	30		
Bwanali et al. (2024) [21]	50		
Wubu et al. (2023) [29]	58		
Agyei‐Baffour et al. (2020) [17]	29		
Venturas and Umeh (2017) [28]	15		
Balogun and Omotade (2018) [18]	190		
Brandt et al. (2019) [20]	41		
Lubeya et al. (2023) [23]	30		
Ochomo et al. (2024) [24]	48		
Rujumba et al. (2021) [25]	40		
Turiho et al. (2017) [27]	105		
Abrefah et al. (2025) [33]	59		
**Total**	**695**	**247**	**1574**

One study utilized just a survey approach for data collection among religious leaders [22], whereas three utilized mixed methods involving a combination of both quantitative and qualitative approaches [19, 26, 31]. The samples include adolescent girls [18, 19, 23, 24, 29, 32, 33], as well as teachers and parents [26]. Others utilized only qualitative data collection techniques [17, 18, 20, 21, 23, 24, 25, 27, 28, 29, 32, 33], including focus group discussions, in‐depth interviews, key informant interviews, and observations. These were conducted among adolescents/school‐aged children [18, 19, 23, 24, 25, 26, 27, 29, 32, 33], and parents/caregivers [18, 19, 21, 24, 25, 27, 32], healthcare workers [17, 25, 27, 28, 30, 31, 32], sexually active women [20], school [18, 19, 25, 27], and community stakeholders [18, 22, 25, 27].

Only three of the included articles introduced intervention, such as pre‐vaccination sensitization [26], HPV vaginal self‐sampling, integration of cervical cancer screening into family planning services, and community education [31], as well as education and sensitization, caregiver counseling, provider recommendations, community awareness, and integration of HPV vaccination into teen clubs [32]. However, majority had no form of intervention before or after data collection.

### 3.2 Quality Appraisal Outcomes

Each of the articles included in this review was found to demonstrate rigorous methodology. Consequently, each of them received an appraisal score of 7, the maximum available score, placing them unanimously in the “above average” quality category (Tables S5–S9).

### 3.3 Study Distribution by Country and Study Setting

**FIGURE 3:**
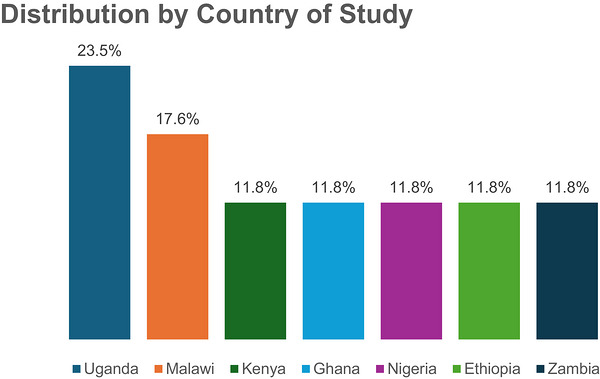
Distribution of reviewed articles by country.

Figure 3 shows that the articles included in this scoping review were distributed across seven African countries: Ethiopia, Ghana, Kenya, Malawi, Nigeria, Uganda, and Zambia. Uganda has the highest number of studies (four), accounting for 23.5% of the total [19, 25, 26, 27], followed by Malawi (17.6%) accounting for three studies [21, 31, 32]. Ethiopia [20, 24], Kenya [22, 24], Nigeria [18, 30], Ghana [17, 33], and Zambia [23, 28] each contribute two studies, representing 11.8% each.

**FIGURE 4:**
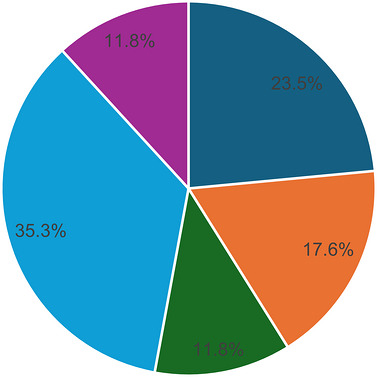
Distribution of included articles by study setting.

As seen in Figure 4, some of the included articles (4/13) were conducted in urban settings only [17, 18, 23, 28], constituting 23.5% of the total. This was followed by articles with samples from rural communities (17.6%) [20, 24, 25]. About 11.8% each were focused on peri/semi‐urban [17, 18] and the sub‐counties [27, 29], respectively. Majority (35.3%) were done in a mixture of settings, including urban and rural, semi‐urban and urban, as well as urban, peri‐urban, and rural samples [19, 26, 30, 31, 32, 33].

### 3.4 Superstitions Concerning HPV and HPV Vaccine

The reviewed articles documented different kinds of superstitions regarding HPV and HPV vaccines, revealing how widespread superstitions are across the region. This scoping review revealed poor community understanding, extensive misinformation, and the influence of cultural and spiritual beliefs, all of which combine to magnify fears and uncertainties regarding the benefits and safety of HPV vaccines across Africa. The superstitions reported in the articles have been classified into different themes as follows (see Table 4).

**TABLE 4:** Distribution of studies reporting specific superstition theme.

Superstition themes	Studies reporting	Number of studies
Myths regarding causation of cervical cancer	Balogun and Omotade [18] Brandt et al. [20] Mhango et al. [31]	3
Supernatural origins and effects of HPV vaccine	Rujumba et al. [25] Lubeya et al. [23]	2
Harmful intent of HPV vaccine	Bitariho et al. [19] Makanani et al. [32] Bwanali et al. [21] Manga et al. [30] Turiho et al. [26] Rujumba et al. [25] Venturas and Umeh [28] Wubu et al. [29] Turiho et al. [27] Lubeya et al. [23] Ochomo et al. [24]	11
HPV vaccination as a cause of other illnesses and premature death	Bitariho et al. [19] Abrefah et al. [33] Bwanali et al. [21] Manga et al. [30] Kaaria et al. [22] Turiho et al. [26] Turiho et al. [27] Rujumba et al. [25] Wubu et al. [29] Ochomo et al. [24] Lubeya et al. [23] Venturas and Umeh [28] Agyei‐Baffour et al. [17]	13

### 3.5 Superstitions Regarding the Causation of Cervical Cancer

Only a few of the studies documented myths regarding the causation of cervical cancer. Among adolescents in Nigeria, there were myths that cervical cancer is caused by curses, wearing trousers by females, poor personal and menstrual hygiene, modern food processing and additives, eating unripe fruits, sweet foods, or meat; modern medicine; early resumption of sexual activity following childbirth [18]; and contraceptives, rather than HPV [18, 31]. In Ethiopia, cervical cancer is traditionally attributed to unexpected body exposure to sunlight (known as Mitat/Girefat), sitting on hot surfaces, and urinating on hot ground [20]. Rather than associating it with cervical cancer, some adolescent girls in Ghana believed HPV could damage the womb and women's ability to give birth and also affect menstruation [33].

### 3.6 Supernatural Origins and Effects of HPV Vaccine

In Uganda, there was the belief that the vaccine may come from supernatural sources such as “satanic agents.” However, some claimed that the HPV vaccine is “for people from under the water” [25]. Similarly, in Zambia, adolescent girls had suspicions that vaccination efforts may involve blood donation for witchcraft activities or satanic rituals [23].

### 3.7 Harmful Intent of HPV Vaccination

In Uganda, Ethiopia, Malawi, and Nigeria, many participants had suspicions that the vaccine was part of the government's plot to kill people or children in mysterious ways as a form of population control [19, 21, 25, 27, 28, 29, 30, 32]. This suspicion was because only young girls were targeted with the vaccine, whereas older females were neglected [27]. Similarly, in Zambia and Kenya, HPV vaccination was seen as a plot by the whites to kill and depopulate women and kill Africans [23, 24].

Some parents and adolescent girls in Uganda also suspected that local politicians had colluded with scientists to administer vaccines that would harm children's health and autonomy [27] and cause slow death in cryptic ways [25].

### 3.8 HPV Vaccination as a Cause of Other Illnesses and Premature Death

People in Uganda believed that HPV vaccine contains harmful substances, implying it was intentionally designed to make people sick. There were claims that the vaccine injects diseases [29], which may cause ailments like HIV [22, 25] or complicate it [27], and could cause cervical cancer [26, 27, 28], which will later result to death of the recipients [26]. Some believed it could cause diabetes, cancer, or other ailments due to some mysterious or hidden properties [25]. In Ethiopia, it was believed that the vaccine could cause blood cancer [29].

In Uganda, there were fears that the vaccine might kill children [25, 27], cause cognitive impairments or reduce mental power in order to render them politically subservient, worsen existing health conditions, predispose the uterus to infections [27], cause strange ailments [24, 27], repeated illness [24], and even death [27], reflecting a mystical attribution of the vaccine side effects.

Majority of the reviewed articles (10/14) documented beliefs regarding the long‐term health effects of HPV vaccine such as indicated by participants in Zambia [23, 28], Nigeria [30], Ghana [33], Ethiopia [29], Malawi [21], Kenya [24], and Uganda [19, 25, 26, 27]. Common belief among people in these locations is that the vaccine was designed to sterilize girls by destroying their ovaries, making them incapable of having children in the future to control the population. Evidently, infertility is highly unacceptable in African cultures such as in Ethiopia [29].

Additionally, in Uganda, HPV vaccine was associated with unnatural disruption of menstrual cycle, childbirth complications [26, 27], and mysterious conception of multiple babies [27]. It is also believed to cause miscarriages [25], cancer [23], and paralysis [23, 24]. In Kenya, parents believed the vaccine would disrupt emotions and sexual health in young girls and make them lose interest in marriage [24]. In Nigeria, there were beliefs linking the vaccine to HPV infection, serious side effects and complications, including autoimmune diseases. Some believed HPV vaccine was too new and not well tested enough to guarantee safety against all diseases [30].

Evidence from the scoping review further suggests that opposition to HPV vaccination by the people was rooted in their strong traditional and religious beliefs. Some community members in Uganda and Ethiopia revealed that HPV vaccination conflicts with their religious and cultural values [27, 29]. There were fears that HPV vaccination will violate their traditional rules or practices, as it disrupts natural or spiritual order in their community [25]. Likewise in Ghana, encouraging HPV vaccination is perceived as immoral and against their religious values [17].

### 3.9 Community Influence and Superstitions About HPV

Some of the studies acknowledged the role of community narratives in spreading fear and superstitions about HPV and its vaccine [25]. Notably, the impact of community elders (specifically old women and custodians of tradition and religion) perpetuating superstitions and misinformation was reported [18, 25].

## 4 Discussion

The current review analyzed the scope of articles on superstitions around HPV in Africa. Seventeen eligible articles were synthesized. The identification of only 17 studies over a decade strongly suggests a genuine and systemic research gap rather than a purely methodological oversight. The limited number of studies reflects chronic challenges across much of the African continent, including significant underinvestment in research funding and limited scientific infrastructure [34, 35]. Furthermore, the topic under review may be marginalized within national and continental research agendas, which often prioritize other fields [36]. A substantial portion of relevant work may also exist outside traditional academic journals—disseminated through grey literature such as NGO reports, theses, or government documents—which is systematically underrepresented in major databases [36]. The review may have been constrained by language filters (e.g., excluding French, Portuguese, or Arabic publications) or database selection (over‐reliance on indexed international databases vs. regional or institutional repositories). However, the low yield is not an artifact of the search strategy but is itself a critical finding. It underscores an urgent need for greater investment in local research and such theme as health‐related superstitions concerning HPV, among others.

The findings show the complexity of cultural beliefs, misinformation, and community narratives surrounding HPV and its vaccine in the region. Extensive fears and superstitions were hindering HPV vaccination efforts, for which traditional/religious heads and cultural norms and values play significant parts in shaping community perceptions and attitudes. Notably, we identified some evidence of misconceptions and non‐HPV attributions of cervical cancer causation among Nigerian and Ethiopian samples [18, 20]. Beliefs that cervical cancer is caused by curses, early sexual encounters following childbirth and exposure to sunlight, and so forth, are of concern because they reflect that cultural narratives still dwarf scientific understanding of HPV in parts of Africa. However, although the evidence strongly suggests that cultural narratives were influential, the included studies did not provide quantitative comparative knowledge assessments to directly measure this effect.

Other studies found myths to be common while knowledge about causation of cervical cancer was poor [37, 38]. Beliefs about spiritual causation rather than HPV, which is primarily responsible for cervical cancer, pose significant public health challenge that could hinder effective prevention and control strategies [39]. Such beliefs have been associated with stigmatization, low rates of screening [39], vaccination uptake [19, 22], and delay in seeking appropriate medical care, as individuals may rely on traditional remedies rather than medical interventions.

Despite the fact that HPV vaccination is central to World Health Organization's cervical cancer control initiative [40], many participants in the reviewed studies believed HPV vaccines contain harmful substances designed to induce illness or sterilize young girls as part of a population control agenda in Africa. In Zambia and Kenya, HPV vaccination is perceived as a tool for depopulation orchestrated by Westerners [23, 24]. This perspective is associated with colonial experience and exploitation and has implications for HPV and other health interventions in Africa as people view them with skepticism and as potential means of population control. Hence, fears and conspiracy theories significantly undermine public acceptance of the initiatives to prevent cervical cancer [41].

In addition, beliefs concerning sterilization have extensive consequences for vaccination initiatives and women's health in general, to such an extent that they are interlinked with African cultural values that prioritize fertility and reproductive health [29]. These misconceptions are particularly prominent because HPV vaccine targets young girls, while older women are not, thus raising questions about its benefits [27, 29].

The scoping review confirms that a significant majority of participants across the region associate the vaccine with unnatural side effects and potential long‐term health consequences. In parts of Africa, misconceptions that HPV vaccines are spiked with harmful substances designed to cause severe health issues such as HIV, diabetes, cancer, or even death were reported among religious leaders, school girls, and caregivers [22, 25, 29]. Additionally, there are fears that HPV vaccine could disrupt natural bodily functions and lead to miscarriages, childbirth complications, or death. In Kenya, parents worry it might affect their daughters’ emotional well‐being and future marital prospects [24]. Similar fears were confirmed regarding COVID‐19 vaccine, which is thought to be unsafe as it could cause serious health challenges, including disruption of the internal make‐up and body functions [42]. This confirms a critical gap in community understanding of the purpose, benefits, and safety of HPV vaccine. Such beliefs hold significant implications for cervical cancer prevention and control in the region where the burden is already high [21, 24].

Furthermore, the belief that the HPV vaccine may originate from supernatural sources was also documented by some reviewed articles, indicating mistrust and suspicions of the health intervention. This reveals social anxieties about novel health interventions, remarkably those perceived as foreign to the people or imposed by outsiders [41]. Due to suspicions, some parents forbade their children from getting vaccinated [24, 27]. A similar experience was reported in the United States, Nigeria, and Lebanon [43, 44, 45, 46, 47].

The findings regarding the role of community narratives in perpetuating fear and superstition about the HPV vaccine reveal the significance of cultural beliefs and traditional authorities in shaping public perception. In some studies, it was observed that community heads, especially older women and custodians of tradition and religion, play a major role in circulating superstitions about HPV vaccine [25]. By endorsing myths rather than factual information, these narratives reflect inherent mistrust in modern medical interventions and the role of traditional authority figures in the efficacy of public health efforts. Traditional and religious heads are significant because they influence individual and community health behaviors [48].

Additionally, the scoping review showed that people in the region had fears concerning the long‐term health complications of HPV vaccination, which is ascribed to mystical or hidden properties of the vaccine. This fear is compounded by existing cultural values that prioritize fertility and reproductive health, making infertility unacceptable [29, 49]. The implications of the community narratives documented in this scoping review are profound. They contribute largely to vaccine hesitancy and lower uptake rates across regions [43, 44, 45, 46, 47]. As cervical cancer remains a leading cause of mortality among women in many African countries [22], failure to address superstitious beliefs could result in preventable deaths and increased healthcare costs associated with late‐stage cancer treatments [43, 44, 45, 46, 47].

Moreover, the intersection of traditional beliefs with modern health programs calls for targeted educational interventions that engage community leaders and address specific cultural concerns. By promoting culturally sensitive dialogue and building trust within communities, public health interventions can effectively neutralize misinformation and superstitions, build trust in vaccination, and promote HPV vaccine acceptance in order to enhance cervical cancer control.

## 5 Limitations

A key limitation of this review concerns both the scope and methodological characteristics of the included evidence. First, the available studies originated from only seven countries, which cannot adequately represent the immense geographic, cultural, and health system diversity of the African continent. Therefore, the findings are not generalizable to all of Africa and should be understood as reflecting the specific contexts from which data were available. Second, most studies employed cross‐sectional or qualitative designs, which restricts our ability to infer causality regarding the relationship between superstitious beliefs and vaccination outcomes. Furthermore, many studies were limited by small sample sizes (see Tables 2 and 3) and single‐site settings, and the frequent use of non‐validated or locally adapted measurement instruments introduces potential bias and limits comparability. Consequently, although this review synthesizes valuable initial insights, the evidence base currently consists of context‐specific findings that are exploratory in nature. The conclusions drawn should be interpreted as indicative of potential associations rather than established causal effects, highlighting the need for more representative, extensive, and methodologically rigorous research.

## 6 Conclusion

This scoping review has provided a comprehensive analysis of the role of superstitions and cultural narratives in shaping perceptions of HPV and its vaccine in Africa. The synthesis of 13 eligible studies highlights the deep‐seated fears, misconceptions, and mistrust that significantly hinder HPV vaccination efforts and cervical cancer prevention strategies in the region. Findings indicate that spiritual attributions, misinformation, and distrust in modern medical interventions contribute to vaccine hesitancy, with traditional and religious leaders playing a crucial role in shaping community beliefs. These misconceptions not only discourage vaccine uptake but also lead to stigmatization, low screening rates, and delays in seeking medical intervention, ultimately worsening the cervical cancer burden in Africa.

The widespread belief that HPV vaccines cause infertility, and chronic diseases, or are part of a Western depopulation agenda reflects a broader skepticism toward public health initiatives. Such skepticism has been reported in several African countries [50, 51, 52]; hence, insights for intervention can be derived and applied beyond the studied countries. Historical events, such as unethical clinical trials, continue to fuel suspicions, making it imperative for public health programs to engage community influencers, religious leaders, and traditional custodians in health education efforts. Given that African cultural values emphasize fertility and reproductive health, interventions should be culturally tailored, trust‐based, and community‐driven to address concerns effectively.

To counter the negative impact of superstitions on HPV vaccine acceptance, there is an urgent need for community‐centered health education, robust public awareness campaigns, and strategic partnerships with local influencers to dispel misinformation. Governments and health organizations must prioritize evidence‐based, culturally sensitive communication strategies to build public confidence in HPV vaccination and cervical cancer prevention programs. Without proactive measures to address these deep‐rooted beliefs, Africa will continue to struggle with high cervical cancer rates and avoidable healthcare burdens. Therefore, tackling superstition and misinformation is not just a medical necessity but a public health imperative.

Specifically, community‐driven education programs must prioritize culturally relevant and participatory strategies. Engaging trusted figures such as religious leaders, traditional healers, and women's group leaders is essential to dismantle fears around infertility, immorality, or spiritual contamination often associated with the vaccine. Community education should be embedded in everyday settings—schools, mosques, markets—and employ storytelling, testimonies, peer education, and visual tools in local languages. Youth ambassadors and mothers’ circles can serve as powerful agents of change, promoting accurate knowledge, addressing sensitive concerns, and reshaping harmful beliefs from within the community. These grassroots engagements not only legitimize biomedical messaging but also provide spaces for dialogue and reflection that challenge myths while respecting local norms.

Complementing these efforts, health communication campaigns must be designed to influence attitudes, not just awareness. Messaging should use relatable themes—protecting daughters’ futures, preparing for motherhood, or maintaining family honor—framed positively to resonate with collective values. Mass media outlets like radio, drama, social media, and mobile vans can extend reach, while myth‐busting IEC materials distributed through clinics and religious centers ensure consistency. Critically, the campaigns should respond directly to specific superstitions, providing clear answers and testimonies that debunk misinformation. Sustained engagement, rather than one‐off efforts, is key—backed by monitoring systems to track perception shifts and guide adaptive messaging. Together, these strategies create a culturally grounded approach to improving HPV vaccine acceptance and reducing the influence of superstition on life‐saving health decisions.

## Author Contributions

Conceptualization, project administration, resources, software, supervision, validation, visualization, supervision, writing – original draft, writing – review and editing: Jimoh Amzat. Conceptualization, data curation, formal analysis, funding acquisition, investigation, methodology, project administration, resources, software, validation, visualization, writing – original draft, writing – review and editing: Kafayat Aminu. Conceptualization, data curation, investigation, methodology, project administration, resources, software, validation, visualization, writing – original draft, writing – review and editing: Rita Amarachi Nwebo. Conceptualization, data curation, investigation, methodology, project administration, resources, software, validation, visualization, writing – original draft, writing – review and editing: Precious Nnannah. Resources, writing – original draft: Chiamaka Norah Ezeagu. Writing – review and editing: Ganiyat Amzat. Conceptualization, data curation, funding acquisition, investigation, methodology, project administration, resources, software, supervision, validation, visualization, writing – original draft, writing – review and editing: Kehinde Kazeem Kanmodi.

## Ethics Statement

The authors have nothing to report.

## Conflicts of Interest

The authors declare no conflicts of interest.

## Transparency Statement

The manuscript guarantor, Kehinde Kazeem Kanmodi, affirms that this manuscript is an honest, accurate, and transparent account of the study being reported; that no important aspects of the study have been omitted; and that any discrepancies from the study as planned (and, if relevant, registered) have been explained.

## Supporting information




**Table S1**. Search string for PubMed database search.
**Table S2**. Search string for SCOPUS database search.
**Table S3**. Search string for other databases (AMED—The Allied and Complementary Medicine Database, CINAHL Ultimate, Dentistry and Oral Sciences Source, SPORTDiscus with Full Text, APA PsycArticles, Psychology and Behavioral Sciences Collection, and APA PsycInfo) search via EBSCO interface.
**Table S4**. List of articles whose full texts were screened for inclusion/exclusion into the scoping review (these are articles obtained from the first round of literature search of research databases).
**Table S5**. Quality appraisal outcomes of the appraised qualitative study using the Mixed Methods Appraisal Tool.
**Table S6**. Quality appraisal outcomes of the appraised quantitative randomized studies using the Mixed Methods Appraisal Tool.
**Table S7**. Quality appraisal outcomes of the appraised quantitative non‐randomized studies using the Mixed Methods Appraisal Tool.
**Table S8**. Quality appraisal outcomes of the appraised quantitative descriptive study using the Mixed Methods Appraisal Tool.
**Table S9**. Quality appraisal outcomes of the appraised mixed‐methods study design using the Mixed Methods Appraisal Tool.

## Data Availability

Data sharing is not applicable to this article as no new data were created or analyzed in this study. All authors have read and approved the final version of the manuscript. Kehinde Kazeem Kanmodi, who is the corresponding author and manuscript guarantor, had full access to all of the data in this study and takes complete responsibility for the integrity of the data and the accuracy of the data analysis.
