# Supplementary material for: Superstitions on Human Papillomavirus in Africa: A Scoping Review
Source: Public Health Chall. 2026 May 21;5(2):e70255. doi: 10.1002/puh2.70255 (PMC13239407; doi:10.1002/puh2.70255)
Supplement: Supplementary file 1 — Table S1. Search string for PubMed database search. Table S2. Search string for SCOPUS database search. Table S3. Search string for other databases (AMED—The Allied and Complementary Medicine Database, CINAHL Ultimate, Dentistry and Oral Sciences Source, SPORTDiscus with Full Text, APA PsycArticles, Psychology and Behavioral Sciences Collection, and APA PsycInfo) search via EBSCO interface. Table S4. List of articles whose full texts were screened for inclusion/exclusion into the scoping review (these are articles obtained from the first round of literature search of research databases). Table S5. Quality appraisal outcomes of the appraised qualitative study using the Mixed Methods Appraisal Tool. Table S6. Quality appraisal outcomes of the appraised quantitative randomized studies using the Mixed Methods Appraisal Tool. Table S7. Quality appraisal outcomes of the appraised quantitative non‐randomized studies using the Mixed Methods Appraisal Tool. Table S8. Quality appraisal outcomes of the appraised quantitative descriptive study using the Mixed Methods Appraisal Tool. Table S9. Quality appraisal outcomes of the appraised mixed‐methods study design using the Mixed Methods Appraisal Tool. [file PUH2-5-e70255-s001.docx]

**SUPPLEMENTARY FILE**

**Table S1. Search string for PubMed database search**

| **Tag** | **Subject search** | **Search String** | **Hits (Updated Search - 16 December 2025)** |
| --- | --- | --- | --- |
| #1 | Superstition | ((((((((superstit*) OR (fallacy)) OR (delusion)) OR (misconception)) OR (fantasy)) OR (falsehood)) OR (falsity)) OR (false notion)) OR (irrational belief) | 44995 |
| #2 | Human papillomavirus | (Human papilloma*) OR (HPV) | 94852 |
| #3 | African countries, dependencies, and territories | (((((((((((((((((((((((((((((((((((((((((((((((((((((((((((Algeria[Title/Abstract]) OR (Angola[Title/Abstract])) OR (Benin[Title/Abstract])) OR (Botswana[Title/Abstract])) OR (burkina faso[Title/Abstract])) OR (burundi[Title/Abstract])) OR (cabo verde[Title/Abstract])) OR (cape verde[Title/Abstract])) OR (cameroon[Title/Abstract])) OR (central african republic[Title/Abstract])) OR (chad[Title/Abstract])) OR (comoros[Title/Abstract])) OR (congo[Title/Abstract])) OR (ivory coast[Title/Abstract])) OR (cote d ivoire[Title/Abstract])) OR (djibouti[Title/Abstract])) OR (democratic republic of congo[Title/Abstract])) OR (egypt[Title/Abstract])) OR (equatorial guinea[Title/Abstract])) OR (eritrea[Title/Abstract])) OR (eswatini[Title/Abstract])) OR (ethiopia[Title/Abstract])) OR (gabon[Title/Abstract])) OR (gambia[Title/Abstract])) OR (ghana[Title/Abstract])) OR (guinea[Title/Abstract])) OR (guinea bissau[Title/Abstract])) OR (kenya[Title/Abstract])) OR (lesotho[Title/Abstract])) OR (liberia[Title/Abstract])) OR (libya[Title/Abstract])) OR (madagascar[Title/Abstract])) OR (malawi[Title/Abstract])) OR (mali[Title/Abstract])) OR (mauritania[Title/Abstract])) OR (mauritius[Title/Abstract])) OR (morocco[Title/Abstract])) OR (mozambique[Title/Abstract])) OR (namibia[Title/Abstract])) OR (niger[Title/Abstract])) OR (nigeria[Title/Abstract])) OR (rwanda[Title/Abstract])) OR (sao tome and principe[Title/Abstract])) OR (senegal[Title/Abstract])) OR (seychelles[Title/Abstract])) OR (sierra leone[Title/Abstract])) OR (somalia[Title/Abstract])) OR (south africa[Title/Abstract])) OR (south sudan[Title/Abstract])) OR (sudan[Title/Abstract])) OR (tanzania[Title/Abstract])) OR (togo[Title/Abstract])) OR (tunisia[Title/Abstract])) OR (uganda[Title/Abstract])) OR (zambia[Title/Abstract])) OR (zimbabwe[Title/Abstract])) OR (reunion[Title/Abstract])) OR (saint helena[Title/Abstract])) OR (western sahara[Title/Abstract])) OR (mayotte[Title/Abstract]) | 496775 |
| #4 | #1 AND #2 AND #3 | ((#1) AND (#2)) AND (#3) | 13 |

**Table S2. Search string for SCOPUS database search**

| **Tag** | **Subject search** | **Search String** | **Hits (Updated Search - 16 December 2025)** |
| --- | --- | --- | --- |
| #1 | Superstition | ( ALL ( superstit* ) OR ALL ( fallacy ) OR ALL ( delusion ) OR ALL ( misconception ) OR ALL ( fantasy ) OR ALL ( falsehood ) OR ALL ( falsity ) OR ALL ( "false notion" ) OR ALL ( "irrational belief" ) ) | 634,290 |
| #2 | Human papillomavirus | ( ALL ( "human papilloma*" ) OR ALL ( hpv ) ) | 284,472 |
| #3 | African countries, dependencies, and territories | ( ( TITLE-ABS-KEY ( angola )  OR  TITLE-ABS-KEY ( benin )  OR  TITLE-ABS-KEY ( botswana )  OR  TITLE-ABS-KEY ( "burkina faso" )  OR  TITLE-ABS-KEY ( burundi )  OR  TITLE-ABS-KEY ( cameroon )  OR  TITLE-ABS-KEY ( "cabo verde" )  OR  TITLE-ABS-KEY ( "cape verde" )  OR  TITLE-ABS-KEY ( "central african republic" )  OR  TITLE-ABS-KEY ( chad )  OR  TITLE-ABS-KEY ( comoros )  OR  TITLE-ABS-KEY ( congo )  OR  TITLE-ABS-KEY ( "ivory coast" )  OR  TITLE-ABS-KEY ( "democratic republic of congo" )  OR  TITLE-ABS-KEY ( djibouti )  OR  TITLE-ABS-KEY ( "equatorial guinea" )  OR  TITLE-ABS-KEY ( eritrea )  OR  TITLE-ABS-KEY ( ethiopia )  OR  TITLE-ABS-KEY ( gabon )  OR  TITLE-ABS-KEY ( gambia )  OR  TITLE-ABS-KEY ( ghana )  OR  TITLE-ABS-KEY ( guinea )  OR  TITLE-ABS-KEY ( guinea-bissau )  OR  TITLE-ABS-KEY ( kenya )  OR  TITLE-ABS-KEY ( lesotho )  OR  TITLE-ABS-KEY ( liberia )  OR  TITLE-ABS-KEY ( madagascar )  OR  TITLE-ABS-KEY ( malawi )  OR  TITLE-ABS-KEY ( mali )  OR  TITLE-ABS-KEY ( mauritania )  OR  TITLE-ABS-KEY ( mauritius )  OR  TITLE-ABS-KEY ( mayotte )  OR  TITLE-ABS-KEY ( mozambique )  OR  TITLE-ABS-KEY ( namibia )  OR  TITLE-ABS-KEY ( niger )  OR  TITLE-ABS-KEY ( nigeria )  OR  TITLE-ABS-KEY ( reunion )  OR  TITLE-ABS-KEY ( rwanda )  OR  TITLE-ABS-KEY ( "saint helena" )  OR  TITLE-ABS-KEY ( sao  AND tome  AND  principe )  OR  TITLE-ABS-KEY ( senegal )  OR  TITLE-ABS-KEY ( seychelles )  OR  TITLE-ABS-KEY ( "sierra leone" )  OR  TITLE-ABS-KEY ( somalia )  OR  TITLE-ABS-KEY ( "south africa" )  OR  TITLE-ABS-KEY ( "south sudan" ) ) )  OR  ( ( TITLE-ABS-KEY ( eswatini )  OR  TITLE-ABS-KEY ( togo )  OR  TITLE-ABS-KEY ( uganda )  OR  TITLE-ABS-KEY ( zambia )  OR  TITLE-ABS-KEY ( zimbabwe )  OR  TITLE-ABS-KEY ( egypt )  OR  TITLE-ABS-KEY ( libya )  OR  TITLE-ABS-KEY ( algeria )  OR  TITLE-ABS-KEY ( tunisia )  OR  TITLE-ABS-KEY ( morocco )  OR  TITLE-ABS-KEY ( "western sahara" )  OR  TITLE-ABS-KEY ( sudan )  OR  TITLE-ABS-KEY ( tunisia ) ) ) | 1,405,034 |
| #4 | #1 AND #2 AND #3 | ( ( ALL ( "human papilloma*" ) OR ALL ( hpv ) ) ) AND ( ( ALL ( superstit* ) OR ALL ( fallacy ) OR ALL ( delusion ) OR ALL ( misconception ) OR ALL ( fantasy ) OR ALL ( falsehood ) OR ALL ( falsity ) OR ALL ( "false notion" ) OR ALL ( "irrational belief" ) ) ) AND ( ( ( ( TITLE-ABS-KEY ( angola ) OR TITLE-ABS-KEY ( benin ) OR TITLE-ABS-KEY ( botswana ) OR TITLE-ABS-KEY ( "burkina faso" ) OR TITLE-ABS-KEY ( burundi ) OR TITLE-ABS-KEY ( cameroon ) OR TITLE-ABS-KEY ( "cabo verde" ) OR TITLE-ABS-KEY ( "cape verde" ) OR TITLE-ABS-KEY ( "central african republic" ) OR TITLE-ABS-KEY ( chad ) OR TITLE-ABS-KEY ( comoros ) OR TITLE-ABS-KEY ( congo ) OR TITLE-ABS-KEY ( "ivory coast" ) OR TITLE-ABS-KEY ( "democratic republic of congo" ) OR TITLE-ABS-KEY ( djibouti ) OR TITLE-ABS-KEY ( "equatorial guinea" ) OR TITLE-ABS-KEY ( eritrea ) OR TITLE-ABS-KEY ( ethiopia ) OR TITLE-ABS-KEY ( gabon ) OR TITLE-ABS-KEY ( gambia ) OR TITLE-ABS-KEY ( ghana ) OR TITLE-ABS-KEY ( guinea ) OR TITLE-ABS-KEY ( guinea-bissau ) OR TITLE-ABS-KEY ( kenya ) OR TITLE-ABS-KEY ( lesotho ) OR TITLE-ABS-KEY ( liberia ) OR TITLE-ABS-KEY ( madagascar ) OR TITLE-ABS-KEY ( malawi ) OR TITLE-ABS-KEY ( mali ) OR TITLE-ABS-KEY ( mauritania ) OR TITLE-ABS-KEY ( mauritius ) OR TITLE-ABS-KEY ( mayotte ) OR TITLE-ABS-KEY ( mozambique ) OR TITLE-ABS-KEY ( namibia ) OR TITLE-ABS-KEY ( niger ) OR TITLE-ABS-KEY ( nigeria ) OR TITLE-ABS-KEY ( reunion ) OR TITLE-ABS-KEY ( rwanda ) OR TITLE-ABS-KEY ( "saint helena" ) OR TITLE-ABS-KEY ( sao AND tome AND principe ) OR TITLE-ABS-KEY ( senegal ) OR TITLE-ABS-KEY ( seychelles ) OR TITLE-ABS-KEY ( "sierra leone" ) OR TITLE-ABS-KEY ( somalia ) OR TITLE-ABS-KEY ( "south africa" ) OR TITLE-ABS-KEY ( "south sudan" ) ) ) OR ( ( TITLE-ABS-KEY ( eswatini ) OR TITLE-ABS-KEY ( togo ) OR TITLE-ABS-KEY ( uganda ) OR TITLE-ABS-KEY ( zambia ) OR TITLE-ABS-KEY ( zimbabwe ) OR TITLE-ABS-KEY ( egypt ) OR TITLE-ABS-KEY ( libya ) OR TITLE-ABS-KEY ( algeria ) OR TITLE-ABS-KEY ( tunisia ) OR TITLE-ABS-KEY ( morocco ) OR TITLE-ABS-KEY ( "western sahara" ) OR TITLE-ABS-KEY ( sudan ) OR TITLE-ABS-KEY ( tunisia ) ) ) ) AND ( ( TITLE-ABS-KEY ( "human papilloma*" ) OR TITLE-ABS-KEY ( hpv ) ) ) AND ( ( TITLE-ABS-KEY ( superstit* ) OR TITLE-ABS-KEY ( fallacy ) OR TITLE-ABS-KEY ( delusion ) OR TITLE-ABS-KEY ( misconception ) OR TITLE-ABS-KEY ( fantasy ) OR TITLE-ABS-KEY ( falsehood ) OR TITLE-ABS-KEY ( falsity ) OR TITLE-ABS-KEY ( "false notion" ) OR TITLE-ABS-KEY ( "irrational belief" ) ) ) ) | 46 |

**Table S3. Search string for other database (AMED – The Allied and Complementary Medicine Database, CINAHL Ultimate, Dentistry and Oral Sciences Source, SPORTDiscus with Full Text, APA PsycArticles, Psychology and Behavioral Sciences Collection, and APA PsycInfo) search via EBSCO interface**

| **Tag** | **Subject search** | **Search String** | **Hits (Updated Search - 13 December 2025)** |
| --- | --- | --- | --- |
| S1 | Superstition | Superstit* OR fallacy OR delusion OR misconception OR fantasy OR falsehood OR falsity OR false notion OR irrational belief | 67,731 |
| S2 | Human papillomavirus | Human papilloma* OR HPV | 19,476 |
| S3 | African countries, dependencies, and territories | AB algeria OR AB angola OR AB benin OR AB botswana OR AB burkina faso OR AB burundi OR AB cape verde OR AB cabo verde OR AB cameroon OR AB central african republic OR AB chad OR AB comoros OR AB congo OR AB cote d'ivoire OR AB ivory coast OR AB djibouti OR AB democratic republic of congo OR AB egypt OR AB equatorial guinea OR AB eritrea OR AB eswatini OR AB ethiopia OR AB gabon OR AB gambia OR AB ghana OR AB guinea OR AB guinea bissau OR AB kenya OR AB lesotho OR AB liberia OR AB libya OR AB madagascar OR AB malawi OR AB mali OR AB mauritania OR AB mauritius OR AB morocco OR AB mozambique OR AB namibia OR AB niger OR AB nigeria OR AB rwanda OR AB ( sao tome and principe ) OR AB senegal OR AB seychelles OR AB sierra leone OR AB somalia OR AB south Africa OR AB south sudan OR AB sudan OR AB tanzania OR AB togo OR AB tunisia OR AB uganda OR AB zambia OR AB zimbabwe OR AB reunion OR AB saint helena OR AB western sahara OR AB mayotte | 163,680 |
| S4 | S1 AND S2 AND S3 | S1 AND S2 AND S3 | 8 |

**Table S4. List of articles whose full texts were screened for inclusion/exclusion into the scoping review (these are articles obtained from the first round of literature search of research databases).**

| **No.** | **CITATION** | **INCLUDED** | **EXCLUDED (REASON)** |
| --- | --- | --- | --- |
| 1 | Agyei-Baffour, P., Asare, M., Lanning, B., Koranteng, A., Millan, C., Commeh, M.E., Montealegre, J.R. and Mamudu, H.M. (2020). Human papillomavirus vaccination practices and perceptions among Ghanaian Healthcare Providers: A qualitative study based on multi-theory model. PLOS ONE, 15(10), p.e0240657. doi:https://doi.org/10.1371/journal.pone.0240657. | Yes |  |
| 2 | Balogun, F. and Omotade, O. (2018). ‘She must have been sleeping around’…: Contextual interpretations of cervical cancer and views regarding HPV vaccination for adolescents in selected communities in Ibadan, Nigeria. PLOS ONE, 13(9), p.e0203950. doi:https://doi.org/10.1371/journal.pone.0203950. | Yes |  |
| 3 | Bitariho, G.K., Tuhebwe, D., Tigaiza, A., Nalugya, A., Tonny Ssekamatte and Kiwanuka, S.N. (2023). Knowledge, perceptions and uptake of human papilloma virus vaccine among adolescent girls in Kampala, Uganda; a mixed-methods school-based study. BMC Pediatrics, [online] 23(1). doi:https://doi.org/10.1186/s12887-023-04174-z. | Yes |  |
| 4 | Brandt, T., Wubneh, S.B., Handebo, S., Debalkie, G., Ayanaw, Y., Alemu, K., Jede, F., von Knebel Doeberitz, M. and Bussmann, H. (2019). Genital self-sampling for HPV-based cervical cancer screening: a qualitative study of preferences and barriers in rural Ethiopia. BMC Public Health, 19(1). doi:https://doi.org/10.1186/s12889-019-7354-4. | Yes |  |
| 5 | Bwanali, A.N., Petro Liundi, Lubanga, A.F., Mpinganjira, S.L. and Gadama, L.A. (2024). Caregiver acceptance of human papillomavirus vaccine for their female children in Chileka, Blantyre, Malawi. Vaccine X, 20, pp.100557–100557. doi:https://doi.org/10.1016/j.jvacx.2024.100557. | Yes |  |
| 6 | Lubeya, M.K., Chibwesha, C.J., Mwanahamuntu, M., Mukosha, M., Frank, S.C. and Kawonga, M. (2023). ‘When you get the HPV vaccine, it will prevent cervical cancer; it will act as a shield’: adolescent girls’ knowledge and perceptions regarding the human papillomavirus vaccine in Zambia. Frontiers in Health Services, [online] 3. doi:https://doi.org/10.3389/frhs.2023.1208458. | Yes |  |
| 7 | Mwansa Ketty Lubeya, Mulindi Mwanahamuntu, Chibwesha, C.J., Mukosha, M. and Kawonga, M. (2024). Selecting and Tailoring Implementation Strategies to Improve Human Papillomavirus Vaccine Uptake in Zambia: A Nominal Group Technique Approach. Vaccines, 12(5), pp.542–542. doi:https://doi.org/10.3390/vaccines12050542. | Yes |  |
| 8 | Ochomo, E.O., Philiph Tonui, Kapten Muthoka, Sayo Amboka, Itsura, P., Elkanah Omenge Orang’o, Rosen, B., Loehrer, P. and Cu-Uvin, S. (2024). Addressing HPV vaccine hesitancy: unveiling concerns and building trust’ perspectives of adolescent girls and parents in Kisumu County, Kenya. ecancermedicalscience, 18. doi:https://doi.org/10.3332/ecancer.2024.1735. | Yes |  |
| 9 | Okenyoru, D.S., Kaaria, F., Odhiambo, F.B., Murugi, L., Matoke, V.O., Salima, R., Anyika, D., Ogutu, G. and Musau, A. (2024). Religious leaders’ willingness to promote the uptake of human papillomavirus vaccine among their congregants in Mavoko Sub-County, Machakos County, Kenya. The Pan African Medical Journal, 48. doi:https://doi.org/10.11604/pamj.2024.48.141.43492. | Yes |  |
| 10 | Otorkpa, O.J., Onifade, A.A. and Otorkpa, C.O. (2024). The Surge in Human Papillomavirus Vaccine Rejection in Nigeria. Cancer prevention research (Philadelphia, Pa.), [online] 17(11), pp.497–498. doi:https://doi.org/10.1158/1940-6207.CAPR-24-0318. |  | Yes (Wrong publication type) |
| 11 | Rujumba, J., Akugizibwe, M., Basta, N.E. and Banura, C. (2021). Why don’t adolescent girls in a rural Uganda district initiate or complete routine 2-dose HPV vaccine series: Perspectives of adolescent girls, their caregivers, healthcare workers, community health workers and teachers. PLOS ONE, 16(6), p.e0253735. doi:https://doi.org/10.1371/journal.pone.0253735. | Yes |  |
| 12 | Tony-Okeke, T.O., Zoakah, J.H., Odoh, A.F., Nnorom, O.C., Aule, S.K., Mathew, M. and Envuladu, E.A. (2024). KNOWLEDGE AND PERCEPTION OF HUMAN PAPILLOMAVIRUS VACCINE AMONG ADOLESCENTS AND CAREGIVERS IN JOS METROPOLIS, PLATEAU STATE, NIGERIA. West African journal of medicine, [online] 41(11 Suppl 1), pp.S47–S48. Available at: https://pubmed.ncbi.nlm.nih.gov/39541268/. |  | Yes (Wrong publication type) |
| 13 | Turiho, A.K., Okello, E.S., Muhwezi, W.W., Harvey, S., Byakika-Kibwika, P., Meya, D. and Katahoire, A.R. (2014). Effect of school-based human papillomavirus (hpv) vaccination on adolescent girls’ knowledge and acceptability of the HPV vaccine in Ibanda District in Uganda. PubMed, 18(4), pp.45–53. | Yes |  |
| 14 | Turiho, A.K., Okello, E.S., Muhwezi, W.W. and Katahoire, A.R. (2017). Perceptions of human papillomavirus vaccination of adolescent schoolgirls in western Uganda and their implications for acceptability of HPV vaccination: a qualitative study. BMC Research Notes, 10(1). doi:https://doi.org/10.1186/s13104-017-2749-8. | Yes |  |
| 15 | Venturas, C. and Umeh, K. (2017). Health professional feedback on HPV vaccination roll-out in a developing country. Vaccine, 35(15), pp.1886–1891. doi:https://doi.org/10.1016/j.vaccine.2017.02.052. | Yes |  |
| 16 | Wubu, A., Balta, B., Cherie, A. and Bizuwork, K. (2023). Perception about human papillomavirus vaccination among middle adolescent school girls in Addis Ababa, Ethiopia 2023: qualitative study. BMC Women’s Health, 23(1). doi:https://doi.org/10.1186/s12905-023-02660-1. |  |  |

**Table S5. Quality appraisal outcomes of the appraised qualitative study using the Mixed Methods Appraisal Tool**

| **No.** | **Author (Year)** | **Study Design** | **Responses to the Appraisal Questions for Qualitative Studies** | | | | | | | **Scored Points (out of a Total of 7 Points)** | **Grade** |
| --- | --- | --- | --- | --- | --- | --- | --- | --- | --- | --- | --- |
|  |  |  | Are there clear research questions? | Do the collected data allow to address the research questions? | Is the qualitative approach appropriate to answer the research question? | Are the qualitative data collection methods adequate to address the research question? | Are the findings adequately derived from the data? | Is the interpretation of results sufficiently substantiated by data? | Is there coherence between qualitative data sources, collection, analysis and interpretation? |  |  |
| 1 | Ochomo et al. (2024) | Qualitative study | Yes | Yes | Yes | Yes | Yes | Yes | Yes | 7 | Above average |
| 2 | Bwanali et al. (2024) | Qualitative study | Yes | Yes | Yes | Yes | Yes | Yes | Yes | 7 | Above average |
| 3 | Brandt et al. (2019) | Qualitative study | Yes | Yes | Yes | Yes | Yes | Yes | Yes | 7 | Above average |
| 4 | Venturas & Umeh (2017) | Qualitative study | Yes | Yes | Yes | Yes | Yes | Yes | Yes | 7 | Above average |
| 5 | Agyei-Baffour et al. (2020) | Qualitative study | Yes | Yes | Yes | Yes | Yes | Yes | Yes | 7 | Above average |
| 6 | Turiho et al. (2017) | Qualitative study | Yes | Yes | Yes | Yes | Yes | Yes | Yes | 7 | Above average |
| 7 | Lubeya et al. (2024) | Qualitative study | Yes | Yes | Yes | Yes | Yes | Yes | Yes | 7 | Above average |
| 8 | Balogun & Omotade (2018) | Qualitative study | Yes | Yes | Yes | Yes | Yes | Yes | Yes | 7 | Above average |
| 9 | Lubeya et al. (2023) | Qualitative study | Yes | Yes | Yes | Yes | Yes | Yes | Yes | 7 | Above average |
| 10 | Rujumba et al. (2021) | Qualitative study | Yes | Yes | Yes | Yes | Yes | Yes | Yes | 7 | Above average |
| 11 | Makanani et al. (2025) | Qualitative study | Yes | Yes | Yes | Yes | Yes | Yes | Yes | 7 | Above average |
| 12 | Abrefah et al. (2025) | Qualitative study | Yes | Yes | Yes | Yes | Yes | Yes | Yes | 7 | Above average |
| Yes – 1 point; No – 0 point; I can’t tell – 0.5 point; Average – 3.5 points; Above average – >3.5 points; Below average – <3.5 points | | | | | | | | | | | |

**Table S6. Quality appraisal outcomes of the appraised quantitative randomized studies using the Mixed Methods Appraisal Tool**

| **No.** | **Author (Year)** | **Study Design** | **Responses to the Appraisal Questions for Quantitative Randomized Studies** | | | | | | | **Scored Points (out of a Total of 7 Points)** | **Grade** |
| --- | --- | --- | --- | --- | --- | --- | --- | --- | --- | --- | --- |
|  |  |  | Are there clear research questions? | Do the collected data allow to address the research questions? | Is randomization appropriately performed? | Are the groups comparable at baseline? | Are there complete outcome data? | Are outcome assessors blinded to the intervention provided? | Did the participants adhere to the assigned intervention? |  |  |
| 1 |  |  |  |  |  |  |  |  |  |  |  |
| Yes – 1 point; No – 0 point; I can’t tell – 0.5 point; Average – 3.5 points; Above average – >3.5 points; Below average – <3.5 points | | | | | | | | | | | |

**Table S7. Quality appraisal outcomes of the appraised quantitative non-randomized studies using the Mixed Methods Appraisal Tool**

| **No.** | **Author (Year)** | **Study**  **Design** | **Responses to the Appraisal Questions for Quantitative Non-randomized Studies** | | | | | | | **Scored Points (out of a Total of 7 Points)** | **Grade** |
| --- | --- | --- | --- | --- | --- | --- | --- | --- | --- | --- | --- |
|  |  |  | Are there clear research questions? | Do the collected data allow to address the research questions? | Are the participants representative of the largest population? | Are measurements appropriate regarding both the outcome and intervention (or exposure)? | Are there complete outcome data? | Are the confounders accounted for in the design and analysis? | During the study period, is the intervention administered (or exposure occurred) as intended? |  |  |
| 1 | Kaaria et al. (2024) | Quantitative analytical study | Yes | Yes | Yes | Yes | Yes | Yes | Yes | 7 | Above average |
| Yes – 1 point; No – 0 point; I can’t tell – 0.5 point; Average – 3.5 points; Above average – >3.5 points; Below average – <3.5 points | | | | | | | | | | | |

**Table S8. Quality appraisal outcomes of the appraised quantitative descriptive study using the Mixed Methods Appraisal Tool**

| **No.** | **Author (Year)** | **Study Design** | **Responses to the Appraisal Questions for Quantitative Descriptive Studies** | | | | | | | **Scored Points (out of a Total of 7 Points)** | **Grade** |
| --- | --- | --- | --- | --- | --- | --- | --- | --- | --- | --- | --- |
|  |  |  | Are there clear research questions? | Do the collected data allow to address the research questions? | Is the sampling strategy relevant to address the research question? | Is the sample representative of the target population? | Are the measurements appropriate? | Is the risk of nonresponse bias low? | Is the statistical analysis appropriate to answer the research question? |  |  |
| 1 | Manga et al. (2025) | Quantitative descriptive study | Yes | Yes | Yes | Yes | Yes | Yes | Yes | 7 | Above average |
| Yes – 1 point; No – 0 point; I can’t tell – 0.5 point; Average – 3.5 points; Above average – >3.5 points; Below average – <3.5 points | | | | | | | | | | | |

**Table S9. Quality appraisal outcomes of the appraised mixed-methods study design using the Mixed Methods Appraisal Tool**

| **No.** | **Author (Year)** | **Study Design** | **Responses to the Appraisal Questions for Mixed-method study designs** | | | | | | | **Scored Points (out of a Total of 7 Points)** | **Grade** |
| --- | --- | --- | --- | --- | --- | --- | --- | --- | --- | --- | --- |
|  |  |  | Are there clear research questions? | Do the collected data allow to address the research questions? | Is there an adequate rationale for using a mixed methods design to address the research question? | Are the different components of the study effectively integrated to answer the research question? | Are the outputs of the integration of qualitative and quantitative components adequately interpreted? | Are divergences and inconsistencies between quantitative and qualitative results adequately addressed? | Do the different components of the study adhere to the quality criteria of each tradition of the methods involved? |  |  |
| 1 | Turiho et al. (2014) | Mixed-methods study | Yes | Yes | Yes | Yes | Yes | Yes | Yes | 7 | Above average |
| 2 | Bitariho et al. (2023) | Mixed-methods study | Yes | Yes | Yes | Yes | Yes | Yes | Yes | 7 | Above average |
| 3 | Mhango et al. (2025) | Mixed-methods study | Yes | Yes | Yes | Yes | Yes | Yes | Yes | 7 | Above average |
| Yes – 1 point; No – 0 point; I can’t tell – 0.5 point; Average – 3.5 points; Above average – >3.5 points; Below average – <3.5 points | | | | | | | | | | | |
